# A rapid acid hydrolysis method for the determination of chitin in fish feed supplemented with black soldier fly (*Hermetia illucens*) larvae

**DOI:** 10.1016/j.heliyon.2022.e09759

**Published:** 2022-06-21

**Authors:** Pedro Araujo, Tamirat Tefera, Joar Breivik, Bashir Abdulkader, Ikram Belghit, Erik-Jan Lock

**Affiliations:** a Norwegian Institute of Marine Research, Feed and Nutrition Group, PO Box 2029, Nordnes, N-5817 Bergen, Norway; b NABAS, Moer Allé 33, 1435 Ås, Norway

**Keywords:** Chitin, Black soldier fly, *Hermetia illucens*, Fish feed, Acid hydrolysis, Liquid chromatography tandem mass spectrometry

## Abstract

Insects are a natural source of feed for fish and have received more attention as a potential source of sustainable high-quality protein. However, contrasting results in different feeding trials have been ascribed to the chitin contained in the exoskeleton of insects and highlighted the importance of developing reliable methods for the quantification of chitin to draw meaningful conclusions about its effect on fish health. A rapid method based on the hydrolysis of chitin into glucosamine and further quantification by liquid chromatography tandem mass spectrometry is evaluated. The method offers good selectivity, linearity, limit of detection (1.08 × 10^−5^ % w/v or 5.38 × 10^−4^ % w/w), limit of quantification (3.26 × 10^−5^ % w/v or 1.63 × 10^−3^ % w/w), trueness (88.39–109.29 %) and precision (2.24–10.72 %). The quantitative method was successfully applied to real samples of fish feed supplemented with chitin from black soldier fly (*Hermetia illucens*) larvae.

## Graphical abstract

**Figure:**
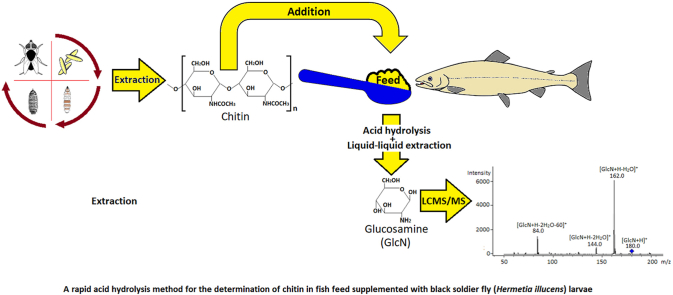

## 1 Introduction

**Figure 1:**
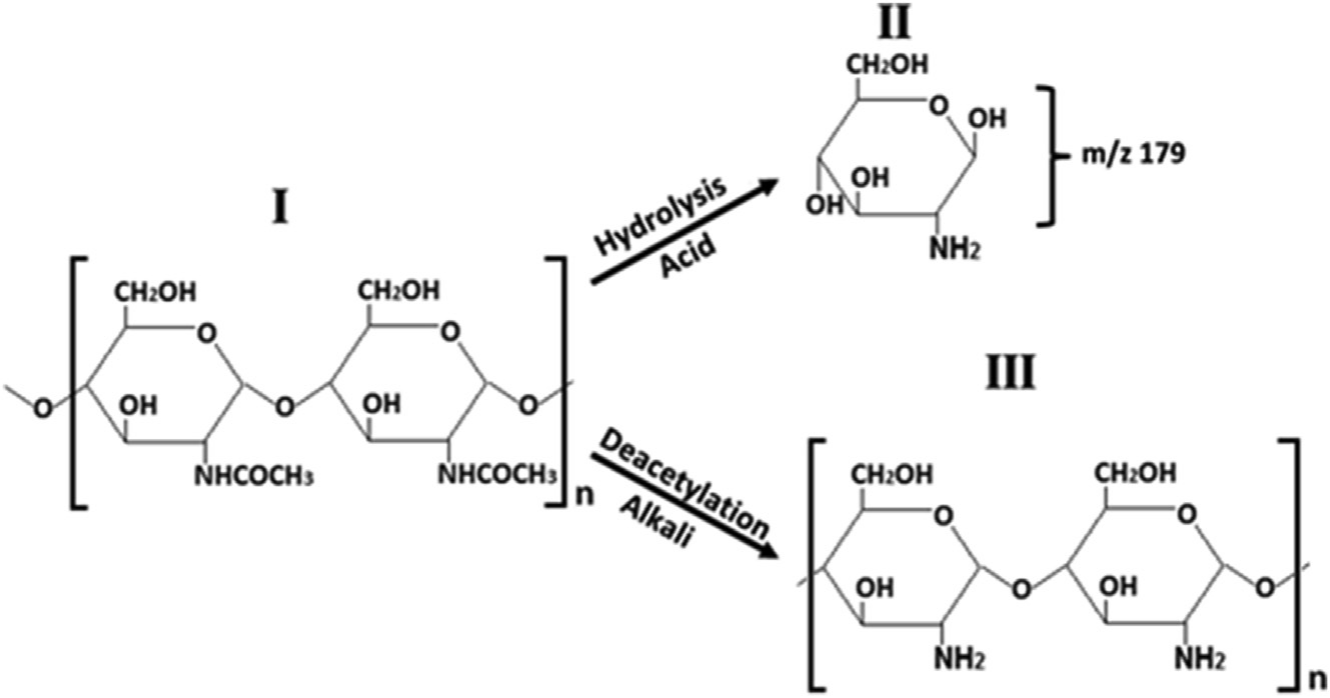
Structure of chitin (I), glucosamine (II) and chitosan (III).

Chitin was discovered over 210 years ago, and since then, its hydrolysis has been studied in different solvents, including alkali and acids (Braconnot, 1810). Chitin is the most abundant natural amino polysaccharide, and it has a molecular structure similar to cellulose but with an acetamide group instead of a hydroxyl group within the glucose unit (Figure 1). Chitin and its derivatives glucosamine (GlcN) and chitosan possess many properties that make them attractive for a wide variety of applications such as food, beverages, nutraceuticals, cosmetics, healthcare, biotechnology, biomedicine, agriculture, and the environment. Increasing demand for chitin and its derivatives will propel the market size that was valued at $42.29 billion in 2020 and is projected to reach $69.297 billion in 2028 (Global Chitin Market Size, 2021).

The aquaculture industry has been striving to implement a sustainable alternative to fishmeal in the fish feed ingredients due to the rising fishmeal price and environmental prospects (OECD/FAO, 2020). Insects are a natural source of feed for fish and have the potential to become a sustainable source of high-quality protein (Alfiko et al., 2021; Rumpold and Schlüter, 2013). Research on new feed ingredients, such as insects, has proliferated recently and will continue to expand, due to their potential for replacing fishmeal and fish oil in aquaculture (Naylor et al., 2021). The value of insects as complete feed or feed ingredients has been established for several fish species in both temperate and tropical climes (Cordis/EU, 2016). For instance, black soldier fly (BSF) (*Hermetia illucens*) has been used in diets for channel catfish (*Ictalurus punctatus*) and blue tilapia (*Oreochromis aureus*) (Bondari and Sheppard, 1987), Rainbow Trout (*Oncorhynchus mykiss*) (Sealey et al., 2011; Renna et al., 2017) and Atlantic salmon (*Salmo salar*) (Belghit et al., 2019). Protein and fat levels of 420 and 350 g/kg in dry matter of prepupae BSF have been estimated with no slaughter waste, consequently the entire larvae can be used as feed ingredient (van Huis and Oonincx, 2017). These features, make BSF one of the most promising insect species for commercial exploitation in Europe (Lock et al., 2016). However, the variability of chitin through different life cycles of BSF (e.g.*,* 7.8 ± 0.3% in larvae, 10.9 ± 0.7% in prepupae, 10.7 ± 0.1% in pupae, 23.7 ± 1.9% in shedding and 22.4 ± 0.9% in cocoon) and its effect on fish feed digestibility and growth constitute one of the most anticipated challenges in the use of insect meal (Soetemans et al., 2020; Danulat, 1987; Santos-Romero et al., 2017; Karlsen et al., 2017).

Fish feeding trials using diets containing chitin have reported contradictory results on growth performance (Tibbetts et al., 2011; Moren et al., 2006; Suontama et al., 2007; Colombo-Hixson et al., 2013; Karlsen et al., 2017). For instance, the study of fish species from the Cyprinidae family revealed that dietary levels of chitin up to 2 % have no effect on growth rate of golden mahseer (*Tor putitore*), while the growth of snowtrout (*Schizothorax richardsonii*) was significantly enhanced at the same levels of chitin (Mohan et al., 2009). These contrasting results might be explained by the lack of chitinolytic enzymes, and/or specific conditions in the digestive tract of the fish, as demonstrated in a study where the hydrolysis of chitin by chitinases was only observed in the guts of cod (*Gadus morhua*) but not in flounder (*Platichthys flesus*) and salmonid (*Coregonus lavaretus*) (Danulat, 1987; Rösch, 1985). The level of chitin inclusion is also another potential factor responsible for the dichotomy of results on digestibility and growth performance. For example, the effect of dietary chitin on nutrient digestibility and growth in farmed Atlantic fish species has revealed that cod and halibut were unaffected by up to 5 % of chitin in the diet, while levels over 1 % had a negative impact on growth and nutrient utilization in salmon (Karlsen et al., 2017). Increasing crab by-products (rich source of chitin) in juvenile cod diets increased growth in a dose-dependent manner (Toppe et al., 2006; Karlsen et al., 2017).

Chitin is degraded to amino sugar GlcN by acid hydrolysis and to chitosan by alkaline deacetylation (Figure 1). These two extensively studied hydrolysis reactions have been used in the development of different procedures to determine chitin in different kind of samples (e.g., water solutions, crab, shrimp, crustacean, molluscan shells, squilla, fossil arthropods, cell walls and chitin standards) by means of the quantification of the products GlcN or chitosan using colorimetry (Katano et al., 2016), liquid chromatography (Crespo et al., 2006), gas chromatography (Holan et al., 1980), pyrolysis-gas chromatography/mass spectrometry (Bierstedt et al., 1998), liquid chromatography mass spectrometry (Crespo et al., 2006;Smets and Van Der Borght, 2021) and infrared spectroscopy (Prabu and Natarajan, 2012).

Liquid chromatography mass spectrometric (LCMS) and colorimetric methods are popular approaches of interest for the aquaculture sector for the determination of glucosamine in different kind of samples containing chitin (Crespo et al., 2006; Katano et al., 2016). However, the main difficulties associated with their implementation in routine analysis are the remarkably time-consuming acid hydrolysis reactions (e.g., 13 h) prior to LCMS analysis (Crespo et al., 2006) and/or the lengthy and laborious procedures (e.g., 12 h acid hydrolysis followed by a chemical reaction between glucosamine and some reagents to form a chromogen) prior to colorimetry assay (Katano et al., 2016). It is surprising that these exceedingly time-consuming methods have been recently regarded as easier and faster approaches for chitin analysis (Tsurkan et al., 2021).

Although, the use of insects is becoming the contemporary solution for replacing fishmeal as a sustainable alternative (Cadinu et al., 2020) and chitin content is among the parameters of interest for the regulatory bodies (Cordis/EU, 2016), a rapid method for the determination of chitin in an insect-based fish feed has not been reported yet. Therefore, the main objective of the present research is to develop and validate a fast acid hydrolysis protocol prior to liquid chromatography tandem mass spectrometry (LCMS/MS) for the analysis of fish feed supplemented with chitin from black soldier fly larvae.

## 2 Materials and methods

### 2.1 Chemicals

Acetonitrile (HPLC grade, ≥99.8 %) and methanol (HPLC grade, ≥99.9 %) were purchased from Honeywell (Seelze, Germany). Hexane (HPLC grade, ≥98 %), chloroform (HPLC grade, ≥99.8 %), and HCl (anhydrous, 37 % v/v) were purchased from Merck KGaA (Darmstadt, Germany). Formic acid (98 %), chitin standard purified from shrimp shells (powder form, C9752) and glucosamine hydrochloride (99 % crystalline) were purchased from Sigma-Aldrich (Oslo, Norway). Ultrapure water from Milli-Q system, Millipore (Milford, MA) was used for the preparation of solvents and solutions.

### 2.2 Samples

The samples of fish feed supplemented with chitin (0 and ∼3 % w/w) from BSF larvae were provided by Protix Biosystems BV (Dongen, The Netherlands).

### 2.3 Analysis of the commercial chitin standard

The estimation of the purity of the commercial chitin standard from shrimp shells (99 %) was carried out by using a highly purified BSF standard (95 %), provided by the Fraunhofer Institute for Interfacial Engineering and Biotechnology IGB (Stuttgart, Germany) and Fourier transform infrared spectroscopy (FTIR) in two different modes. Briefly, FTIR in attenuated total reflectance mode was used to determine the similarity between the structures of the commercial and purified BSF standard, and diffuse reflectance mode was used to determine the concentration of chitin in the commercial standard by means of the standard addition method and multivariate calibration models. A detailed description of the determination is provided elsewhere (Gezahegn, 2018).

### 2.4 Evaluation of the acid hydrolysis variables

Preliminary experiments aiming at selecting the optimal hydrolysis conditions that yield the maximum experimental response (chromatographic peak area) were performed using a chitin standard and a full factorial design for four variables at two levels (−1 and +1). The studied variables were: HCl concentration (6 and 12 M), HCl volume (0.5 and 1.0 mL), reaction temperature (80 and 95 °C), and reaction time (30 or 60 min). The chromatographic peak area of GlcN was measured in ion counts per seconds (icps) units. The experimental 2^4^-factorial arrangement is shown in Table 1.

**Table 1:** The 2^4^-factorial design used to study the influence of some of the variables that influence the hydrolysis of chitin into glucosamine (GlcN). The experimental points were prepared in triplicate and the chromatographic peak areas of GlcN were measured in ion counts per seconds (icps) units.

Exp. #	Natural variables				Coded variables				Experimental response		
	HCl		Hydrolysis reaction		var_1_	var_2_	var_3_	var_4_	Chromatographic peak area (icps)		
	Concentration (M)	Volume (mL)	Temperature (°C)	Time (min)					y_1_	y_2_	y_3_
1	6	0.5	85	30	-1	-1	-1	-1	46077	40067	51286
2	12	0.5	85	30	1	-1	-1	-1	54748	58819	47197
3	6	1.0	85	30	-1	1	-1	-1	18722	18897	15747
4	12	1.0	85	30	1	1	-1	-1	369841	308565	364107
5	6	0.5	95	30	-1	-1	1	-1	70691	84203	84123
6	12	0.5	95	30	1	-1	1	-1	415909	409561	353070
7	6	1.0	95	30	-1	1	1	-1	69436	60379	76681
8	12	1.0	95	30	1	1	1	-1	776411	806547	652446
9	6	0.5	85	60	-1	-1	-1	1	50583	44041	52850
10	12	0.5	85	60	1	-1	-1	1	146668	141820	121213
11	6	1.0	85	60	-1	1	-1	1	407002	318444	378948
12	12	1.0	85	60	1	1	-1	1	90625	78804	99952
13	6	0.5	95	60	-1	-1	1	1	99022	98952	84634
14	12	0.5	95	60	1	-1	1	1	250423	198043	237652
15	6	1.0	95	60	-1	1	1	1	68387	87648	80012
16	12	1.0	95	60	1	1	1	1	1329140	1076850	1249145

### 2.5 Hydrolysis of chitin and extraction of glucosamine

The experiments were conducted as follows: 20 mg of fish feed supplemented with chitin from a commercial standard (0.45 %w/w chitin) were mixed with an appropriate volume (0.5 or 1 ml) and concentration (6 or 12M) of HCl. The mixture was then heated (80 °C or 95 °C) for 30 or 60 min. An aliquot of 200 μL of the hydrolysate was collected and dried (Labconco vacuum drier system, Kansas, MO, USA) at room temperature. The dried product was resuspended in 1 mL of methanol, vortex-mixed and subsequent aliquots of hexane (1 mL) and H_2_O (200 μL) were added to the mixture. The mixture was vortex-mixed, centrifuged at 1580 g for 1 min and the hexane phase is removed. The addition of hexane and H_2_O (1 mL and 200 μL, respectively) to the methanol phase and further removal of the hexane were repeated twice. The polar phase was filtered (Chromabond® PE filter 730163, Teknolab, Ski, Norway), dried under a gentle flow of nitrogen, resuspended in 200 μL of water:acetonitrile (63:37 v/v) containing 0.2 % formic acid, centrifuged and submitted to LCMS/MS analysis.

### 2.6 Effect of the methanol/hexane miscibility on the extraction of glucosamine

After hydrolysis of a supplemented sample (∼88 %w/w), an aliquot (200 μL) of the GlcN solution was dried and dissolved in subsequent aliquots of methanol and hexane (1 mL each). The hexane phase is collected, and the procedure is repeated twice by adding only hexane (1 mL) on the initial methanol phase. The collected non-polar hexane phase is dried and redissolved in 200 μL of the mobile phase for injection in the LCMS/MS system.

### 2.7 Instrumental

Agilent 1100 series LC/MSD trap, SL model equipped with an electrospray interface (ESI) was used for the LCMS analysis. The column used was a Zorbax Eclipse-C8 RP 150 mm × 4.6 mm, 5 μm (Agilent Technologies, Palo Alto, CA, USA) kept at 40 °C and the injection volume was 25 μL. The mobile phase in isocratic mode consisted of water:acetonitrile (63:37 % v/v) with formic acid (0.2 % v/v) and delivered at a flow rate of 0.3 mL/min. The total analysis time was 15 min. The ESI source was operated in positive ion mode and nitrogen was used as nebulizing and drying gas at 350 °C. Complete system control, data acquisition and processing were done using the ChemStation for LC-MSD Trap Software, Version 5.3 from © Agilent Technologies, Inc., 2005. The extracted ion chromatogram (EIC) of glucosamine (GlcN) was detected as the protonated adduct [GlcN + H]^+^ at 180 m/z along with its corresponding transitions [GlcN + H–H_2_O]^+^ at 162 m/z [GlcN + H–2H_2_O]^+^ at 144 m/z and [GlcN + H–2H_2_O-60]^+^ at 84 m/z. The EIC were recorded in ion counts per seconds (icps).

### 2.8 Method validation

The EURACHEM recommendations (Magnusson and Örnemark, 2014) were considered for validation purposes. The analytical characteristics evaluated, after selecting the optimal hydrolysis/extraction conditions, were selectivity, linearity, limit of detection (LOD), limit of quantification (LOQ), trueness and precision.

The selectivity of the method was assessed by comparing the GlcN chromatograms of fish feed samples without and with chitin supplementation in triplicates. The linearity of the method was evaluated by using fish feed supplemented with chitin from a commercial standard at seven levels of concentration (0.50, 2.50, 12.5, 25.00, 50.00, 75.00 and 87.50 % w/w) as follows: triplicate aliquots of 20 mg of each supplemented fish feed were dissolved in 1 mL of HCl to get concentrations of chitin of 0.01, 0.05, 0.25, 0.50, 1.00, 1.50, and 1.75 % w/v. Linear regression analysis was applied to fit the model that explains the relationship between peak area and the concentration of chitin in % w/v and % w/w. The LOD and LOQ were estimated by hydrolyzing replicate samples (*n* = 6) of a fish feed without chitin supplementation and calculating the standard deviation (*σ*_*blank*_) of the recorded signals. The EURACHEM guidance suggests the use of *σ*_*blank*_ and the slope (*m*) of the calibration curve as a valid approach to determine the LOD (3.3×*σ*_*blank*_/*m*) and LOQ (10×*σ*_*blank*_/*m*) as reported elsewhere (Shrivastava and Gupta, 2011). The trueness was expressed as the quotient between back-calculated and nominal concentration. The precision was expressed as the quotient between the standard deviation and average concentration (aka coefficient of variation) at each concentration level (0.01–1.75 % w/v or 0.5–87.5 % w/w).

### 2.9 Analysis of supplemented-chitin diets

Four experimental diets were prepared in triplicate by taking aliquots of 20 mg of sample that were submitted to the above-described protocols, consisting of acid hydrolysis → liquid-liquid extraction → LCMS/MS analysis. The experimental diets (designated as A, C, D) consisted of three supplemented fish feed samples containing approximately the same amount of chitin (around 3 % w/w) from BSF larvae, in addition to an extra chitin-free diet (designated as B). These experimental diets were part of a research intended to investigate the effect of including a BSF larvae meal on growth, digestibility, nutrient utilization, liver health and fillet sensory qualities of Atlantic salmon (*Salmo salar*). The information about the chitin concentration in the four samples was withheld from the analyst who received the samples labelled as A, B, C and D.

### 2.10 Data analysis

The regression models were constructed by using an Excel-based macro approach that allows to visualize the calibration curve and residual plot after introducing the nominal concentrations and corresponding recorded signals (Supplementary Excel file S1). The main feature of the Excel-based macro is that it can detect automatically whether a regression model is linear based on the ratio lack-of-fit to pure error variance (aka Fisher test), instead of using the commonly accepted and incorrect approach of the closeness of the coefficient of determination (*r*^*2*^) to a value of one as the only indicator of linearity. Therefore, both values (Fisher test and *r*^*2*^) were considered in the present research to judge the linearity of the model. The macro also provides some characteristic parameters of a calibration such as the slope, the intercept, the *r*^*2*^, the degrees of freedom, the theoretical and experimental Fisher values (*F*_*theoretical*_ and *F*_*experimental*_, respectively), in addition to the calculated trueness and precision.

A *t*-test was performed (*p* = 0.05) to evaluate the differences between the nominal and experimental chitin concentration in the fish feed samples containing approximately the same amount of chitin (around 3 % w/w) from BSF larvae. After checking the normality and homoscedasticity of the data, a Dunnett test (*p* = 0.05) was carried out to evaluate simultaneously significant differences between diets A, C and D.

## 3 Results

### 3.1 Selection of the hydrolysis and extraction conditions

**Figure 2:**
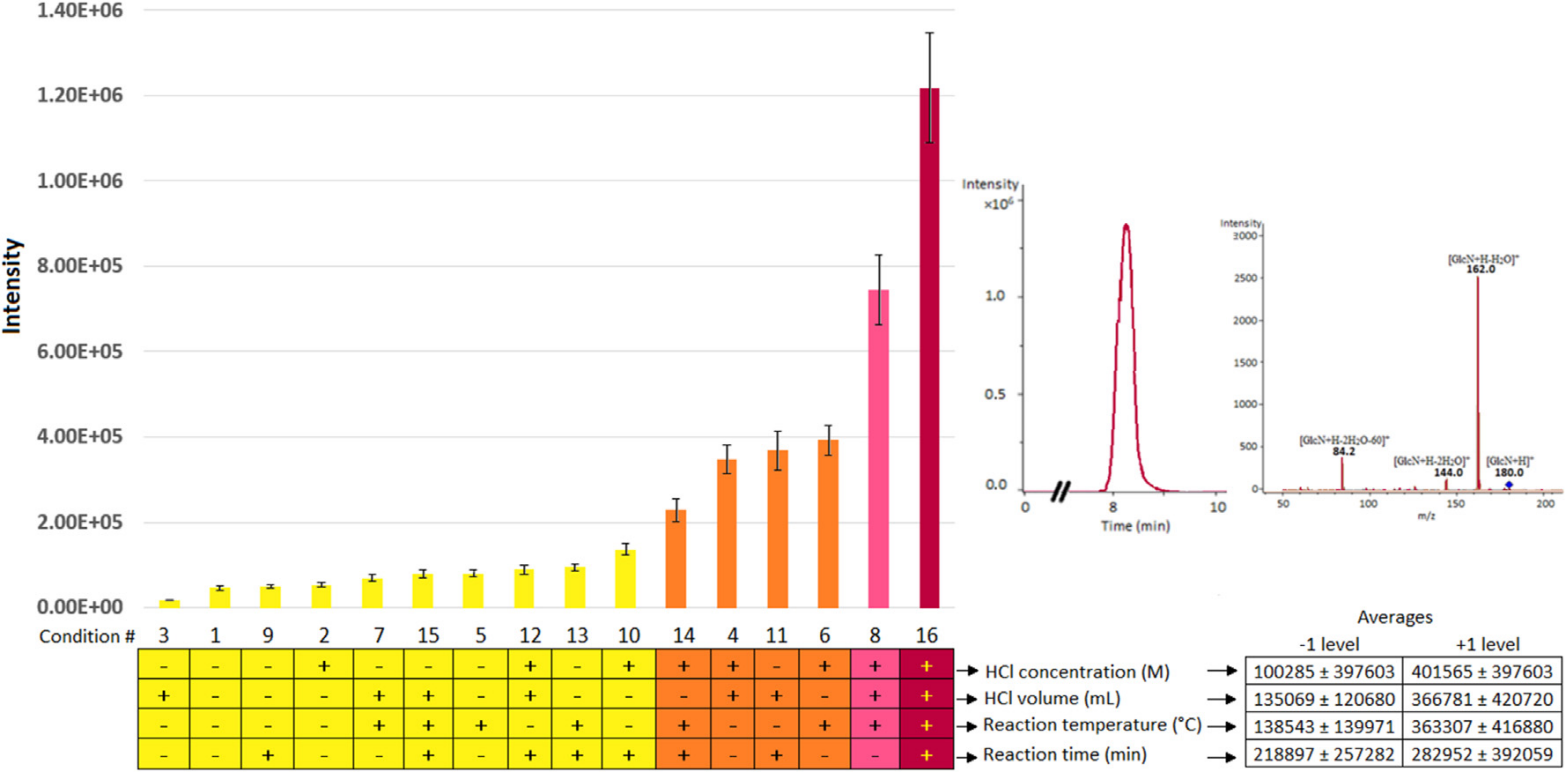
Bar diagram showing the intensity of the GlcN signals recorded under the experimental conditions dictated by the 2^4^-factorial design in Table 1. The coded levels (-1 and +1) of the studied variables are indicated under the bar diagram. The extracted ion chromatogram (EIC) recorded at condition #16 was obtained by the summation of the transitions 162, 144 and 84 m/z from the precursor [GlcN + H]^+^ at 180 m/z and indicated in the mass spectrum. The impact of the factors at the -1 and +1 level is reported as average ±standard deviation (*n* = 3).

The evaluation of the factors that affect the conversion of chitin into glucosamine (Table 1) revealed that the highest conversion of chitin into GlcN was achieved under experimental condition #16 (Figure 2), namely: 12 M HCl, 1 mL HCl, 95 °C and 60 min. The extracted ion chromatogram (EIC) and mass spectrum of GlcN at the experimental condition #16 are shown in Figure 2. The impact of keeping the factors at their low or high experimental magnitude was also investigated by averaging the recorded signals in Table 1 at the specific -1 or +1 level (Figure 2) as follows: the average and standard deviation of the effect of the HCl concentration (100285 ± 397603 M in Figure 2) at -1 level is obtained by averaging the eight signals in Table 1 that were recorded under conditions with odd numbers (#1, #3, #5, #7, #9, #11, #13 and #15), while those conditions with even numbers (#2, #4, #6, #8, #10, #12, #14 and #16) are used to compute the average effect of HCl concentration (401565 ± 397603 M in Figure 2) at +1 level. Similarly, the first eight conditions in Table 1 (#1 to #8) are used to calculate the effect of the reaction time (218897 ± 257282 min in Figure 2) at the -1 level and conditions #9 to #16 to calculate the effect (282952 ± 392059 min in Figure 2) at the +1 level. The average signals of the four factors at the +1 level were consistently higher than the -1 level (Figure 2).

### 3.2 Effect of the methanol/hexane miscibility on the extraction

**Figure 3:**
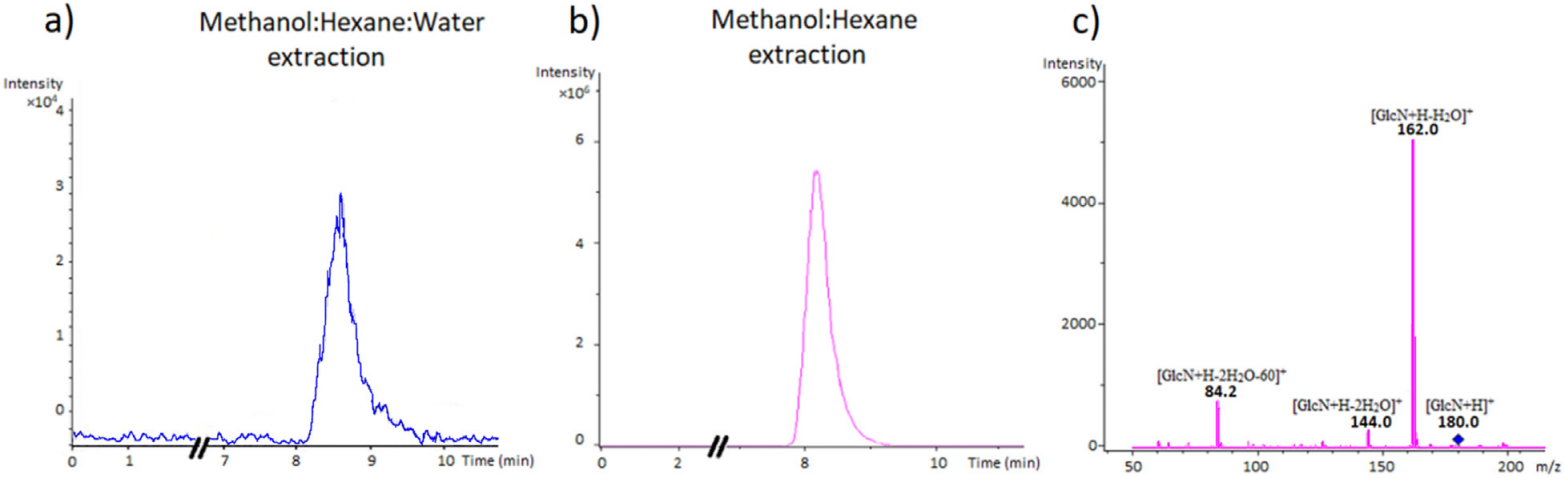
Extracted ion chromatograms (EICs) of the hexane phase after acid hydrolysis of chitin and extraction of glucosamine (GlcN) with (a) and without (b) water. The EICs were obtained by the summation of the transitions 162, 144 and 84 m/z. Tandem mass spectrum of GlcN (without water) indicating the precursor [GlcN + H]^+^ at 180 m/z and its characteristic transitions (c).

It was noted, after adding hexane to the methanol containing the GlcN, that the volumes of the biphasic system methanol/hexane were not equivalent to 1 mL each (methanol phase > hexane phase). Hence, an extra experiment was conducted where the three aliquots of hexane (1 mL each) were kept in the system with methanol and collected after adding 200 μL of water only in the final extraction step, which enable to recover approximately 3 mL of hexane. The collected hexane is dried, redissolved in the mobile phase (200 μL) and injected in the LCMS/MS system. The extracted ion chromatograms of the GlcN (with and without water) obtained after hydrolysis of chitin in 1 mL of HCl (12 M) at 95 °C for 60 min and the corresponding mass spectrum for the precursor [GlcN + H]^+^ and its transitions [GlcN + H–H_2_O]^+^ [GlcN + H–2H_2_O]^+^ and [GlcN + H–2H_2_O-60]^+^ at 180, 162, 144 and 84 m/z, are shown in Figure 3a-c, respectively.

### 3.3 Method validation

The fish feed samples supplemented with chitin were submitted to the above-described optimal hydrolysis conditions and further liquid-liquid extraction of GlcN. The validation parameters selectivity, linearity, LOD, LOQ, trueness and precision were determined by means of LCMS/MS.

The GlcN (180 m/z) and its characteristic fragments (162, 144 and 84 m/z) were detected at a retention time of 8.27 min with no further interferences from the sample matrix.

Calibration curves were constructed by summing up the peak areas of the fragments 162, 144 and 84 m/z from the EIC over the range 0.01–1,75 % w/v. The recorded areas (*y*) as function of the concentrations of GlcN (*x*) are shown in Table 2 and were used to generate the model of the form *y* = *mx* + *b*. The experimental responses and the regression parameters slope (*m* = 6.66×10^7^ icps/w/v% or 1.33×10^6^ icps/w/w%) and intercept (*b* = 1.17×10^6^ icps) were used to estimate the lack-of-fit to pure experimental variance ratio (*F*_*experimental*_ = 1.211) with 5 and 14 degrees of freedom (DF), respectively. The coefficient of determination (*r*^*2*^) of the regression model was 0.998. The statistical ranges for trueness and precision were 88.39–109.29 % and 2.24–10.72 % with median values of 99.82 % and 2.38 %, respectively. The LOD and LOQ were 1.08 × 10^−5^ and 3.26 × 10^−5^ % w/v (5.38 × 10^−4^ and 1.63 × 10^−3^ % w/w), respectively.

**Table 2:** Testing the linearity of the model $\hat{y}=mx+b$ for fish feed supplemented with chitin according to the premise *F*_*experimental*_ < *F*_*theoretical*_ at the 95 % confidence level and the specified degrees of freedom for lack-of-fit and pure errors.

Chitin percentage (*x*)		Experimental area (y)		Estimate area as $\hat{y}=mx+b$	Average experimental area ($\bar{y}$)	Square errors	
						Pure-experimental (*pε*) as ${\left(y-\bar{y}\right)}^{2}$	Lack-of-fit (*lof*) as ${\left(\hat{y}-\bar{y}\right)}^{2}$
(% w/w)	(% w/v)	(icps)		(icps)	(icps)	(icps^2^)	(icps^2^)
0.50	0.01	1.76×10^6^		1.84×10^6^	1.84×10^6^	6.28×10^9^	3.68×10^6^
		1.86×10^6^		1.84×10^6^	1.84×10^6^	3.71×10^8^	3.68×10^6^
		1.90×10^6^		1.84×10^6^	1.84×10^6^	3.60×10^9^	3.68×10^6^
2.50	0.05	4.52×10^6^		4.50×10^6^	4.47×10^6^	2.48×10^9^	8.51×10^8^
		4.69×10^6^		4.50×10^6^	4.47×10^6^	4.71×10^10^	8.51×10^8^
		4.21×10^6^		4.50×10^6^	4.47×10^6^	7.12×10^10^	8.51×10^8^
12.50	0.25	1.68×10^7^		1.78×10^7^	1.72×10^7^	1.57×10^11^	4.45×10^11^
		1.75×10^7^		1.78×10^7^	1.72×10^7^	1.31×10^11^	4.45×10^11^
		1.72×10^7^		1.78×10^7^	1.72×10^7^	1.17×10^9^	4.45×10^11^
25.0	0.50	3.35×10^7^		3.45×10^7^	3.43×10^7^	6.27×10^11^	1.97×10^10^
		3.51×10^7^		3.45×10^7^	3.43×10^7^	5.23×10^11^	1.97×10^10^
		3.44×10^7^		3.45×10^7^	3.43×10^7^	4.67×10^9^	1.97×10^10^
50.0	1.00	6.71×10^7^		6.78×10^7^	6.87×10^7^	2.51×10^12^	8.33×10^11^
		7.01×10^7^		6.78×10^7^	6.87×10^7^	2.09×10^12^	8.33×10^11^
		6.88×10^7^		6.78×10^7^	6.87×10^7^	1.87×10^10^	8.33×10^11^
75.0	1.50	1.01×10^8^		1.01×10^8^	1.03×10^8^	5.64×10^12^	3.87×10^12^
		1.05×10^8^		1.01×10^8^	1.03×10^8^	4.71×10^12^	3.87×10^12^
		1.03×10^8^		1.01×10^8^	1.03×10^8^	4.20×10^10^	3.87×10^12^
87.5	1.75	1.17×10^8^		1.18×10^8^	1.16×10^8^	3.22×10^12^	4.29×10^12^
		1.10×10^8^		1.18×10^8^	1.16×10^8^	3.13×10^13^	4.29×10^12^
		1.19×10^8^		1.18×10^8^	1.16×10^8^	1.45×10^13^	4.29×10^12^
			Sum square errors (SSE) →			6.56×10^13^	2.84×10^13^
			Degrees of freedom (DF) →			14	5
			Variances (SSE/DF) →			4.68×10^12^	5.67×10^12^
			F_experimental_ = (SSE/DF)_lof_/(SSE/DF)_pε_ →				1.211
			F_theoretical_ →				2.958

The values of m and b are 6.66×10^7^ and 1.17×10^6^ in icps/w/w % and icps units, respectively.

The values of m and b are 1.33×10^6^ and 1.17×10^6^ in icps/w/v % and icps units, respectively.

### 3.4 Analysis of supplemented-chitin diets

The levels of chitin in the samples designated as A, B, C and D were 3.32 ± 0.04, non-detected, 3.06 ± 0.09 and 2.95 ± 0.09% w/w, respectively. The *t*-test revealed statistically significant differences only between diet A and a nominal concentration of 3.00 % w/w (*t*_experimental_ = 10.31 versus *t*_theoretical_ = 3.18). The simultaneous comparison of diets A, C and D confirmed effectively that diet A is statistically different from diets C and D (*p* < 0.05).

## 4 Discussion

### 4.1 Selected extraction parameters

The results in Table 1 and Figure 2 suggested that the maximum GlcN signals are obtained by keeping the factors HCl concentration (12 M), HCl volume (1 mL), reaction temperature (95 °C) and reaction time (60 min) at their maximum studied magnitudes (+1 levels of the 2^4^-factorial design). The averages of the recorded signals at -1 and at +1 level were calculated for every factor and the ratio between them (+1/-1) revealed that the average signals at +1 level were 4.0, 2.7, 2.6 and 1.3 times higher than at -1 level for HCl concentration, HCl volume, reaction time and reaction temperature, respectively. These results give more assurance that the selected experimental condition #16, with all the factors at the +1 level, gives the best responses.

### 4.2 Hydrolysis and extraction

A comparison of different acid hydrolysis methods used for the determination of chitin by LCMS is presented in Table 3. Previous LCMS methods recommend hydrolyzing 100 mg of a sample containing chitin in 2 mL of HCl (6 M) for 12–13 h at 100 °C for complete hydrolysis and the use of a large dilution volume (25 mL H_2_O) after hydrolysis completion prior to LCMS analysis (Crespo et al., 2006; Katano et al., 2016). A recent article suggests hydrolyzing a lower amount of sample (20 mg) in 10 mL of HCL (8 M) at 110 °C for 4 h followed by filtration, dilution, drying of the hydrolysate with a stream of nitrogen, re-dissolution in the mobile phase solvents and final 50-fold aqueous dilution before LCMS/MS determination (Nurfikari and de Boer 2021). It is evident that the evaporation of an aqueous solution of HCl (>>10 mL) is a lengthy procedure. In addition, the suggested final dilution will negatively affect the quantification process of samples containing low levels of chitin.

**Table 3:** Comparison of different acid hydrolysis methods for the determination of chitin by LCMS.

Sample (mg)	Experimental conditions				Neutralization after hydrolysis	Derivatization	Reference
	HCl (mol/L)	Volume (mL)	Time (h)	Temperature (°C)			
100	6	2	13	100			Crespo et al. (2006)
20	6	10	4	110			Nurfikari and de Boer (2021)
10	5	10	12	100	✓	✓	Katano et al. (2016)
20	8	10	2	100		✓	Smets and Van Der Borght (2021)
20	12	1	1	95			This work

Similarly, published colorimetric methods for the determination of chitin are based on the implementation of lengthy and complex procedures such as acid hydrolysis (5 M HCl, 12 h) of the sample (10 mg) in a large volume of acid (10 mL) followed by neutralization with sodium hydroxide and the addition of sodium metasilicate, sodium molybdate, acetic acid and dimethyl sulfoxide solution to the resulting hydrolysate to promote the reduction of molybdenum (VI) by GlcN and form the blue molybdosilicate anion with absorbance maximum at 750 nm (Katano et al., 2016). Other published derivatization protocols achieve the hydrolysis of 20 mg of sample at 100 °C in a shorter time (2 h) by using 10 mL of HCl at a higher concentration (8 M) and without the need of neutralization with sodium hydroxide (Smets and Van Der Borght, 2021). The present less complex study uses a higher concentration of HCl (12 M) to hydrolyze 20 mg of sample by using a lower amount of acid (1 mL) to achieve the complete hydrolysis within a substantially shorter time (1 h) and without diluting the final hydrolysate prior to LCMS analysis.

The visually observed lack of equivalence between the volumes of hexane and methanol (methanol phase > hexane phase) during the extraction is the result of the partial miscibility of hexane in methanol that causes a significant reduction in the volume of the hexane layer (and an apparent increasing in the volume of the methanol phase). The addition of water increases the polarity of the water/methanol mixture, decreases the solubility of hexane in methanol and restates the original volume of hexane (Araujo et al., 2016). The previous observations are confirmed by comparing the chromatographic signals with and without water (Figures 3a-b) that cause a higher signal intensity in the hexane phase when the water was avoided (Figure 3b). The observed lower intensity of GlcN in the hexane system after adding water (Figure 3a) could be explained by the presence of the amino group in the GlcN that ionizes in aqueous solutions to form the ${\text{NH}}_{3}^{+}$ which readily interacts with the polar water molecules and increases the affinity of GlcN for the methanol:water phase (Heller et al., 2010).

### 4.3 Method validation

The analysis of the extracted ion chromatograms of fish feed samples supplemented with and without chitin revealed that the chromatographic peak of GlcN and characteristic mass fragmentation patterns were clearly determined without interference from other components in the matrix of the sample, indicating that the analysis is highly selective towards GlcN.

The linearity of the method was not judged by the proximity of *r*^*2*^ to a numerical value of one, as incorrectly assumed in published chitin related literature (e.g., Crespo et al., 2006). The magnitude of *r*^*2*^ should not be used to demonstrate a causation relationship between the recorded signals (*y*) and the concentrations of chitin (*x*). The calculated *r*^*2*^ of 0.998 indicated that a very low percentage of the variance (0.2 %) of the dependent *y*-variable remains as residual and cannot be explained by the independent *x*-variable. The comparison of the experimental and tabulated Fisher ratios (*F*_*experimental*_ = 1.211 and *F*_*tabulated*_ = 2.958) at a 95 % confidence level and 5 and 14 DF (Table 2) was used to establish the linearity of the calibration model on the premise that *F*_*experimental*_ < *F*_*tabulated*_. Consequently, the computed calibration model *y* = 6.66 × 10^7^*x* + 1.17×10^6^ is adequate to quantify chitin according to the proposed experimental protocol.

In general, the present results outperformed similar works using acid hydrolysis and LCMS/MS in terms of amount of sample (20 mg), HCl volume (1 mL), hydrolysis time (1 h), analysis of a small fraction of the hydrolysate (0.2 mL) without dilution, simple procedures prior LCMS/MS and very low LOD (5.38 × 10^−4^ % w/w or 1.08 × 10^−5^ % w/v) and LOQ (1.63 × 10^−3^ % w/w or 3.26 × 10^−5^ % w/v). For instance, some authors have reported the hydrolysis of same amount of sample (20 mg) with larger volumes of HCl (10 mL), longer hydrolysis times (2 h), larger fraction of hydrolysate (1 mL) that is diluted in an exceedingly large volume of water (25 mL), higher LOD (0.25 ng/μL or 2.5 × 10^−5^ % w/v) and LOQ (0.85 ng/μL or 8.5 × 10^−5^ % w/v) values than the present research, and the use of 9-fluorenylmethyl chloroformate as derivatization reagent for GlcN prior to LCMS/MS quantification, which increase the complexity of this recent published method (Smets and Van Der Borght, 2021). Other authors have reported 13 h acid hydrolysis of larger amounts of sample (100 mg) in 2 mL of HCl and LOD of 1.00 × 10^−3^ % w/v (Crespo et al., 2006).

Most studies evaluating the impact of chitin-supplemented diets on fish digestibility and performance are generally focused on levels over 1 % w/w of chitin inclusion as reflected in a review on the use of chitin in aquaculture (Ringø et al., 2012), and also in a meta-analysis on the nutritional value of insects in aquafeeds that states levels over 2.7 % w/w of dried insect meal from BSF (Liland et al., 2021). Hence the proposed protocol with a quantification range over 0.5 % w/w can be regarded as a suitable experimental approach to quantify chitin in insect-based fish feed. In addition, the very low LOD and LOQ values of 5.38 × 10^−4^ and 1.63 × 10^−3^ % w/w (1.08 × 10^−5^ and 3.26 × 10^−5^ % w/v, respectively) may account for the potentiality of the method to quantify chitin in fish organs.

### 4.4 Analysis of supplemented-chitin diets

Information on whether the analyzed three fish feed were supplemented with the same batch of BSF larvae or prepared by the same operator was not available. This preanalytical factors could contribute to explain the observed differences of around 8 and 12 % between diets A&C and A&D, respectively. However, the results of the four experimental diets confirmed the validity of the method for evaluating the impact of chitin on fish digestibility and growth performance, that is generally carried out at levels of chitin supplementation over 1 % w/w. In addition, the results account for the sensitivity of the method to quantify reported natural levels of chitin (8–24 % w/w) through the different life cycles of BSF (Soetemans et al., 2020) or those reported in aquafeeds (2.7–22.6 % w/w) (Liland et al., 2021).

## 5 Conclusions

The proposed protocol for quantifying chitin in an insect-based fish feed is faster and less labour-intensive compared to previously reported procedures by using LCMS/MS.

The validated protocol can determine the analytical ranges considered in aquaculture feeding trials, where the impact of chitin on digestibility and performance is traditionally evaluated over a concentration level of 1 %. Hence, the proposed methodology can be regarded as a suitable strategy to study the effect of supplementing dietary fish meal with chitin from BSF. In addition, the LOD and LOQ are solid baselines that pave the way for the development of risk assessment methodologies for quantification of chitin in fish organs.

Norway and the European Union are committed to gather knowledge on insects for food and feed (The Norwegian Food Safety Authority, 2018) and chitin is an economically valuable by-product of insect farming (Huet et al., 2020). Consequently, the proposed methodology is a promising tool to face the circular economy paradigms in response to sustainability, production scenarios and environmental issues.

**Supplementary excel file S1**. Excel-based macro used to build the regression models in % w/w or % w/v. The macro shows the calibration curve and residual plot after introducing the nominal concentrations and corresponding recorded signals and detects automatically whether a regression model is linear based on the ratio lack-of-fit to pure error variance (aka Fisher test).

## Declarations

### Author contribution statement

Pedro Araujo, Ikram Belghit, Erik-Jan Lock: Conceived and designed the experiments; Analyzed and interpreted the data; Contributed reagents, materials, analysis tools or data; Wrote the paper.

Tamirat Tefera: Performed the experiments; Analyzed and interpreted the data; Wrote the paper.

Joar Breivik, Bashir Abdulkader: Performed the experiments; Analyzed and interpreted the data.

### Funding statement

This work was supported by the Norwegian Research Council through the projects Circularity (grant number: 15456–06) and Aquafly (grant number: 238997), the Institute of Marine Research (IMR), Bergen, Norway. Tamirat Tefera was supported by the integrated international study programme Erasmus Mundus EMQAL (European Joint Master in Quality in Analytical Laboratories).

### Data availability statement

Data included in article/supplementary material/referenced in article.

### Declaration of interests statement

The authors declare no conflict of interest.

### Additional information

No additional information is available for this paper.

## Appendix A Supplementary data

SUPPLEMENTARY EXCEL FILE S1 SUPPLEMENTARY EXCEL FILE S1
